# Case Report: A *Chlamydia psittaci* pulmonary infection presenting with migratory infiltrates

**DOI:** 10.3389/fpubh.2022.1028989

**Published:** 2022-12-19

**Authors:** Jundi Wang, Yurou Zhu, Qiongya Mo, Yanfei Yang

**Affiliations:** ^1^Department of Rheumatology, Affiliated Hangzhou First People's Hospital, Zhejiang University School of Medicine, Hangzhou, Zhejiang, China; ^2^Department of Respiratory Diseases, Hangzhou TCM Hospital Affiliated to Zhejiang Chinese Medical University, Hangzhou, Zhejiang, China

**Keywords:** *Chlamydia psittaci*, migratory infiltrates, CT, mNGS, pneumonia

## Abstract

Community-acquired pneumonia is a public health problem in all countries in the world, with a broad range of causative agents and *Chlamydia psittaci* infection tends to be overlooked. Pulmonary migratory infiltrates are commonly seen in eosinophilic pneumonia, cryptogenic organizing pneumonia, etc. However, the association of *Chlamydia psittaci* and pulmonary migratory infiltrates has been seldom described in literatures before. We reviewed a 64-year-old man referred to our hospital for treatment against *Chlamydia psittaci* pneumonia which was diagnosed by metagenomics next generation sequencing (mNGS). During the treatment period, chest imaging showed migratory infiltrates, which has been rarely described before.

## Introduction

The first report of the human-to-human transmission of *Chlamydia psittaci* was documented by Zhenjie Zhang in China in 2020 (1). Pulmonary migratory infiltrates are commonly seen in eosinophilic granulomatosis with polyangiitis (EGPA), cryptogenic organizing pneumonia and so on Tiralongo et al. (2), the imaging manifestations are intrapulmonary infiltrative lesions, no fixed lung segment or lobe distribution, and intrapulmonary migratory shadow. However, the association of *Chlamydia psittaci* pulmonary infection and migratory infiltrates has been never described in literatures before.

Patients suffering from severe *Chlamydia psittaci* infection are prone to sepsis and multiple organ failure. The difficulty in identifying psittacosis with traditional detection methods usually leads to misdiagnosis of this condition in clinical practice (3), because before metagenomic next-generation sequencing (mNGS), the main method for scientists to diagnose *Chlamydia psittaci* infection was to notice an elevation of chlamydia-specific IgG antibodies in serological assays, and its convalescent serum increased four-fold (4). It's an approach of retrospective as we all know. Within the global scope, mNGS develops so fast with the unprecedented speed as to improve the speed and accuracy of clinic diagnosis (5). We herein describe a patient with *Chlamydia psittaci* pulmonary infection diagnosed by bronchoalveolar lavage fluid mNGS who presented with migratory infiltrates, and be cured after antimicrobial therapy.

## Case presentation

**Figure 1:**
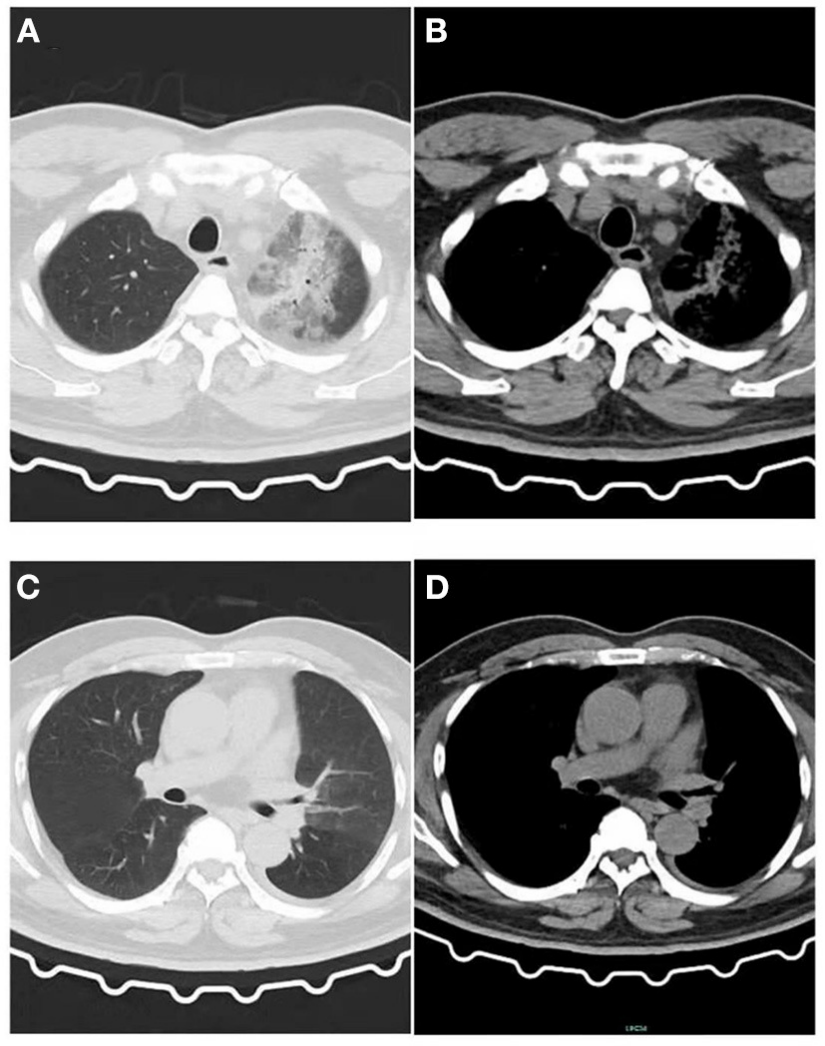
Image of the patient's first CT inspection on June 5, 2021. **(A)** Lung window: Patchy high-density shadow in the upper lobe of the left lung with blurred edges. **(B)** Mediastinal window: Patchy exudative lesions in the upper lobe of the left lung. **(C)** Lung window: No obvious exudative lesions in the left lower lobe, and a small amount of pleural effusion on the left. **(D)** Mediastinal window: No obvious exudative lesions.

**Figure 2:**
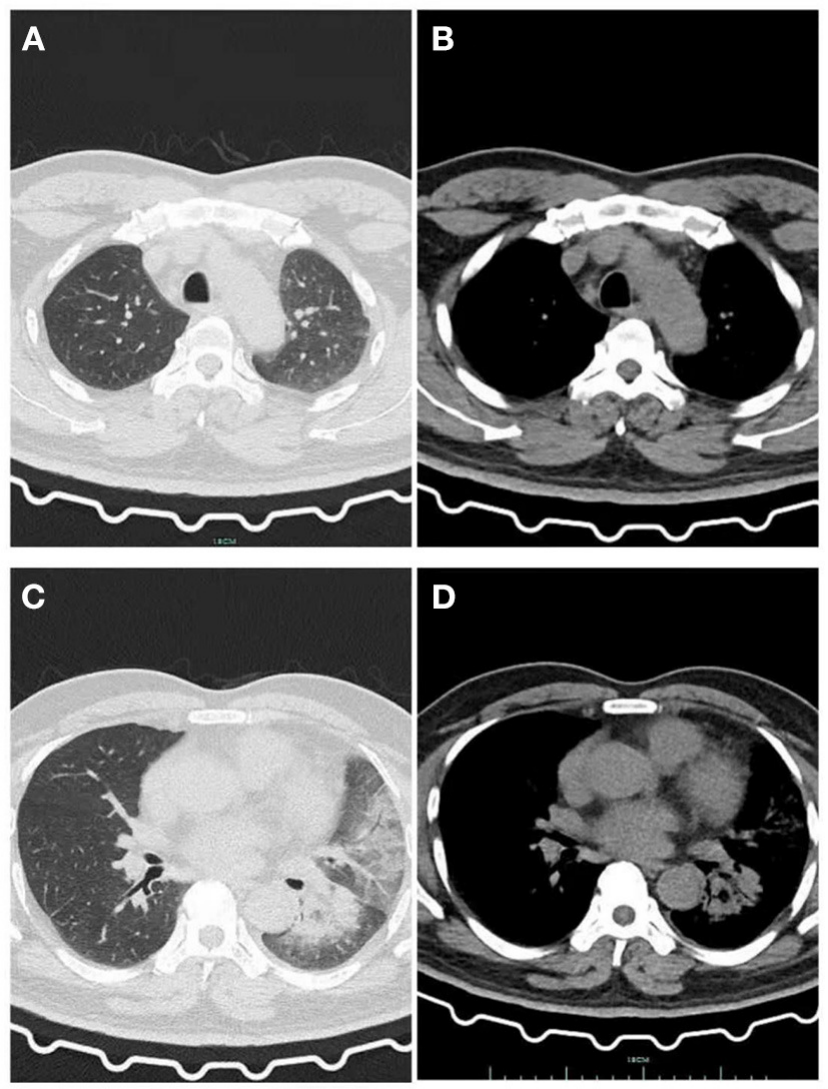
After he was given Latamoxef Sodium as empirical treatment for 6 days. **(A)** Lung window: The patchy high-density shadow in the upper lobe of the left lung is obviously absorbed. **(B)** Mediastinal window: Exudative lesions in the upper lobe of the left lung were obviously absorbed. **(C)** Lung window: New patchy high-density shadow in the lower lobe of the left lung with i blurred edges, pleural effusion was absorbed more than before. **(D)** Mediastinal window: Newly emerging exudative lesions in the left lower lobe.

**Figure 3:**
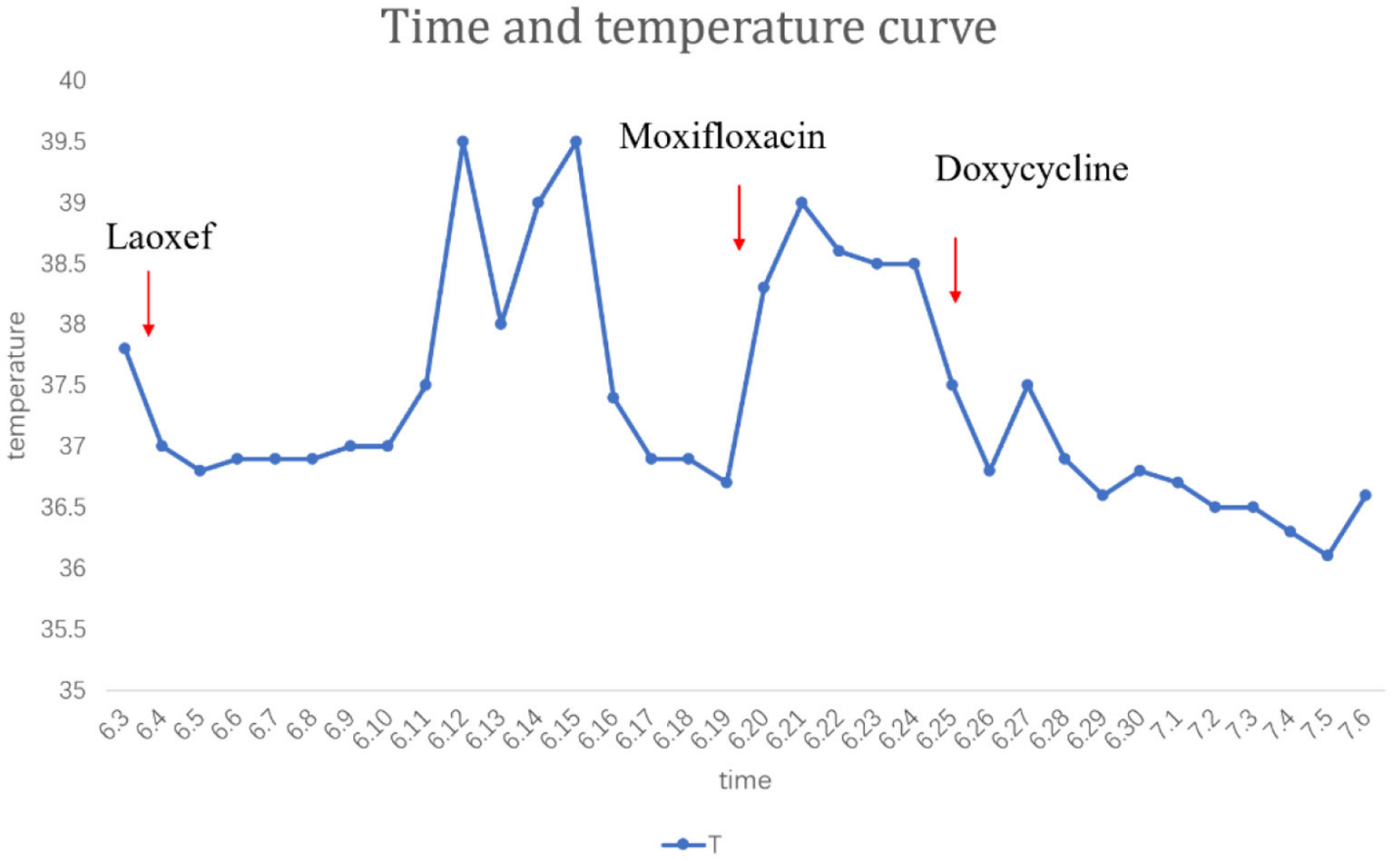
The temperature during his treatment.

**Figure 4:**
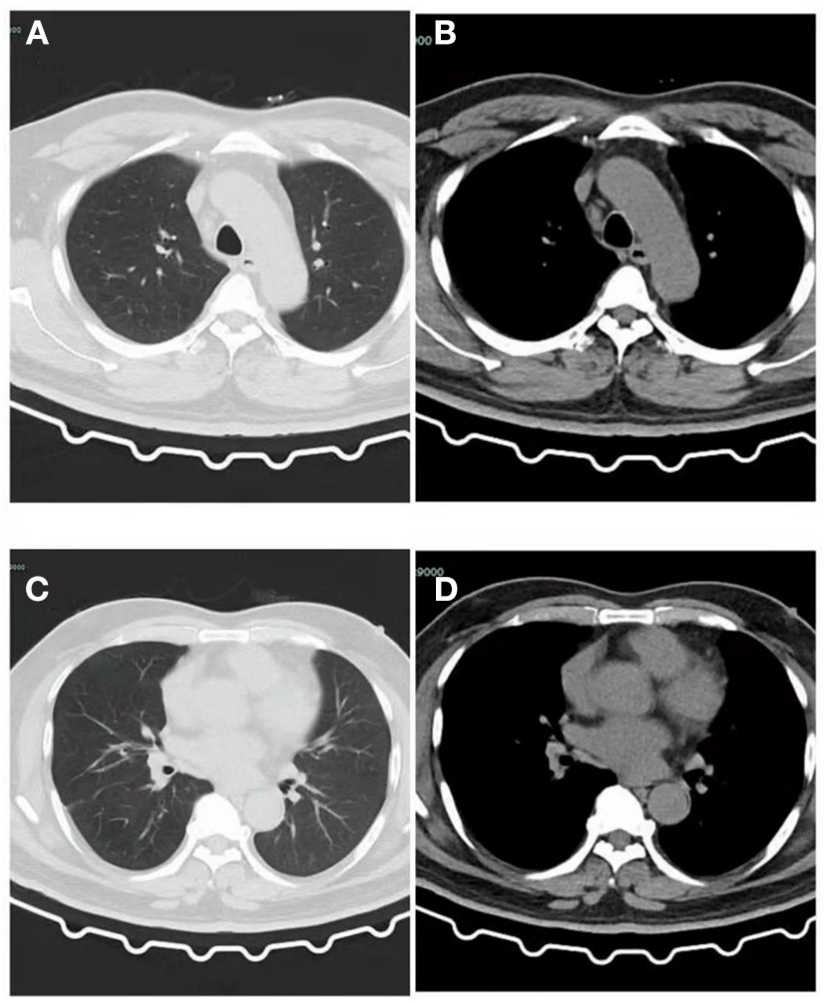
CT of the patient after recovery. **(A)** Lung window: The patchy high-density shadow in the upper lobe of the left lung have been completely absorbed, and a few cord-like shadows can be seen with clear edges. **(B)** Mediastinal window: No obvious exudative lesions in the left upper lobe. **(C)** Lung window: The patchy high-density shadow and pleural effusion in the lower lobe of the left lung have been completely absorbed, and a few cord-like shadows can be seen with clear edges. **(D)** Mediastinal window: No obvious exudative lesions in the left lower lobe.

A 64-year-old male patient was admitted to our hospital on June 5, 2021. He was a 64-year-old retired public health worker. Following his retirement, He owned a non-commercial chicken-farms. He presented to our hospital with the chief complaint of fever and cough. Initially, he was diagnosed with bacterial pneumonia with 3 weeks of low-grade fever. CT revealed exudative lesions on the left upper lung field at the onset of disease (Figure 1). Latamoxef Sodium—a broad-spectrum cephalosporin—was given intravenously as empirical treatment in this patient for the treatment as the patient had been misdiagnosed as bacterial pneumonia at first. During treatment, the patient complained of intermittent cough and chest congestion. Before transferred to our department, he was evaluated by computed tomography (CT) scan again, which indicated a new lesion of consolidation in the left lower lobe (Figure 2). During the period following his referral, he had a continuous cough and a high fever of 39.5°C (Figure 3). Chest imaging showed migratory infiltrates. During detailed medical history taking, we noticed that the patient had exposure to chicken manure. Thus, we highly suspect that there could potentially be infected by atypical infections included cryptococcal infection or *Chlamydia psittaci*. To fight against atypical pathogens (mostly the Chlamydia spp), the patient was empirically treated with Moxifloxacin (400 mg/d) intravenously which resulted in an improvement of clinical symptoms. In parallel, we performed bronchoscopy for this patient whose bronchoalveolar lavage fluid were collected for mNGS. Although his serum was negative for anti- *Chlamydia psittaci* antibody, mNGS of his bronchoalveolar lavage fluid (BALF) showed only the presence of *Chlamydia psittaci* pneumophila(The mNGS data indicated 16 sequences aligning to *Chlamydia psittaci)*, and eosinophils and white blood cell counts in routine blood tests were normal. So the treatment with Doxycycline(100 mg q12 h)was instituted for him. Assuredly, the treatment improved his cough and sputum, as well as his pulmonary lesions (Figure 4). However, we are still confused about the CT imaging results which was characterized by fleeting infiltrates.

## Discussion and conclusions

In our case, the patient had a clear contact history of chicken manure. We suspect that his exposure to *Chlamydia psittaci* might be derived from the chicken manure because he had a big chicken farm. Notwithstanding, there were no features of the spread of *Chlamydia psittaci* pneumonia among his family. Although it has previously been reported that Chlamydia has the potential for human-to-human transmission (1, 6). This can partly be explained by the known epidemiology of *Chlamydia psittaci* in humans in that most human cases are a result of animal-to-human transmission, rather than human-to-human transmission. Since there are 10 genotypes of *Chlamydia psittaci* (7), with varying preference for host species, further study is necessary to determine the genetic diversity of *Chlamydia psittaci*.

Normally, *Chlamydia psittaci* pneumonia shows certain characteristics, including high fever, cough, fatigue, etc. CT imaging often shows different degrees of exudation and patchy consolidation, unilateral involvement is more common, and the white blood cell count can be normal (8). Molecular diagnostic methods such as mNGS could lead to rapid diagnosis and treatment which can shorten the course of hospitalization and thus improve prognosis (9). In our case, mNGS provides basis for the accurate diagnosis of *Chlamydia psittaci* (The mNGS data indicated 16 sequences aligning to *Chlamydia psittaci)*. Doxycycline-based therapy is effective in severe *Chlamydia psittaci* pneumonia. Failure to diagnose the disease or identify the pathogen in time, with delayed use of effective antibiotics can cause high mortality in severe cases (10).

*Chlamydia psittaci* infection tends to be overlooked due to relatively low awareness by physicians. The current case series indicated that mNGS could be used to diagnose *Chlamydia psittaci* infection (7). Considering the high cost of mNGS test, we recommend the early use of mNGS testing in failure cases of empirical treatment and severe cases. In our case, the patient had a history of direct exposure to chicken manure. When the patient had persistent fever even after the use of empiric antibiotic therapy, the patient received mNGS analysis of bronchoalveolar lavage fluid (BALF) and mNGS clearly and unambiguously detected 16 sequences for *Chlamydia psittaci* in BALF. In accordance with previous results by mNGS, *Chlamydia psittaci* pulmonary infection was determined. The patient received doxycycline and moxifloxacin with a successful therapeutic response. However, until now, rarely literature has described the association of pulmonary migratory infiltrates with *Chlamydia psittaci* pulmonary infection. The migratory characteristic of these infiltrates can be seen in chronic eosinophilic pneumonia (CEP) (11), ANCA-associated vasculitis, including granulomatosis with polyangiitis, eosinophilic granulomatosis with polyangiitis, and microscopic polyangiitis (12). Previous studies revealed that a case of pulmonary migratory infiltrates (PMI) attributed to *mycoplasma pneumonia (Mp)* infection (4). In our case, The imaging manifestations of pulmonary migratory infiltrates attributed to *Chlamydia psittaci* infection can be rare. To our knowledge, there are rare reports of such cases. It has already been reported that organizing pneumonia was also appeared in some cases of atypical organisms' infection (13). These findings can be seen as non-specific reactions to injury and also occur in association with some infections. We suspect that infectious agents such as *Chlamydia psittaci* may have caused some cases of COP and some of them may have shown migratory pulmonary infiltrates (and some of them recovered without steroid therapy) (14). We suspect that similar circumstance was in our case. However, we have never used steroids, the lesion has completely disappeared. Of course, it could also be a combined bacterial infection, but mNGS did not provide a basis of bacterial infection.

In conclusion, the present study described an extremely rare case of *Chlamydia psittaci* pulmonary infection, which was initially misdiagnosed as bacterial pneumonia. Patients described overall satisfaction with the treatment, after all, we have reduced invasive examination on patient such as Lung puncture and so on. If the patient does not respond to treatment, an alternative diagnosis should be considered and invasive procedures should be performed to provide an accurate diagnosis. We call for mNGS examination for patients with migratory pulmonary infiltrates, not just a lung puncture in those patients (previously, migratory pneumonia was mainly considered as non-infectious organized pneumonia). More than anything, CT of the chest may show migratory infiltrates in *Chlamydia psittaci* infection.

## Data availability statement

The original contributions presented in the study are included in the article/supplementary material, further inquiries can be directed to the corresponding author.

## Ethics statement

Written informed consent was obtained from the individual(s) for the publication of any potentially identifiable images or data included in this article.

## Author contributions

Conception, design, collection, and assembly of data: YY. Data analysis and interpretation: YZ and QM. Manuscript writing: JW and YY. Final approval of manuscript: All authors.
